# P2X7 protein expression and polymorphism in non-small cell lung cancer (NSCLC)

**DOI:** 10.1186/1477-5751-13-16

**Published:** 2014-09-01

**Authors:** Laura Boldrini, Mirella Giordano, Greta Alì, Adele Servadio, Serena Pelliccioni, Cristina Niccoli, Alfredo Mussi, Gabriella Fontanini

**Affiliations:** 1 Department of Surgical, Medical, Molecular Pathology and Critical Area, University of Pisa, Via Roma 57, 56126 Pisa, Italy; 2 Unit of Pathological Anatomy III, A.O.U.P., 56126 Pisa, Italy

**Keywords:** P2X7, Polymorphism, NSCLC

## Abstract

**Background:**

P2X7, a purinergic receptor, plays important roles in inflammatory diseases, but recently its expression has been found in several tumors, suggesting a potential role as a cancer cell biomarker. Moreover, the relative amount of P2X7 varies among human individuals due to numerous single nucleotide polymorphisms resulting in either a loss- or gain-of-function; the P2X7 gene is highly polymorphic, and polymorphisms in the promoter or coding region may modify its expression or function. A polymorphism in exon 13 of the P2X7 receptor gene at the +1513 position (Glu496Ala substitution, corresponding to SNP rs3751143) has been shown to eradicate the function of this receptor and has been correlated with histological variants and clinical parameters in thyroid cancer. Until now, no data regarding P2X7 expression and polymorphisms in lung cancer have been published; based on these premises, we decided to evaluate the impact of the P2X7 expression and polymorphisms in ninety-seven cases of non-small cell lung cancer (NSCLC).

**Results:**

No significant difference in the genotype frequency of the A1513C polymorphism was found between the two histological variants of NSCLC, adenocarcinoma and squamous cell carcinoma, and no statistically significant associations were observed between P2X7 protein expression and the main clinico-pathological characteristics of the NSCLC patients.

**Conclusions:**

Based on our results, P2X7 expression and polymorphisms seem to have no potential impact in patients with non-small cell lung cancer; however, further studies will surely provide deeper insights regarding the role of this receptor at the clinical level in NSCLC.

## Background

ATP-gated P2X7 receptors (P2X7) comprise a unique family of extracellular ATP-activated plasma membrane ion channels expressed in hematopoietic and epithelial cells. P2X7 receptors have been extensively studied in immune cells where their activation leads to the rapid release of pro-inflammatory cytokines and the initiation of the inflammatory cascade. As such, P2X7 represent a pharmaceutical target for the treatment of inflammatory diseases. Recently, P2X7 expression has been found in several types of tumors [1-5], and P2X7 expression has been suggested as a potential cancer cell biomarker. Upon ATP stimulation, tumor cells can use P2X7 signaling in different scenarios: i) as a reaction to this death-related signal, tumor cells can downregulate P2X7 to avoid apoptosis, or ii) as a cancer-promoting signal, P2X7 signaling can promote survival and enhanced invasion of new niches. The high levels of extracellular ATP found in tumors could represent a stressful stimulus for cancer cells by initiating P2X7-driven cell death. Therefore, the increased P2X7-dependent invasiveness of cancer cells could be an escape strategy to flee the noxiously high levels of ATP. The use of specific P2X7 antagonists could be a new, alternative way to reduce the development of cancer metastases and improve the efficacy of conventional treatments [6].

The P2X7 gene is highly polymorphic, and polymorphisms in the promoter or coding region may modify its expression or function. A polymorphism in exon 13 of the P2X7 receptor gene at the +1513 position (Glu496Ala substitution, corresponding to SNP rs3751143) has been shown to eradicate the function of this receptor and has been correlated with histological variants and clinical parameters in thyroid cancer [7]. Until now, no data existed regarding P2X7 expression and polymorphisms in lung cancer; Fernando et al. reported that subjects that carry one or two copies of SNP rs3751143 exhibit an enhanced susceptibility to extrapulmonary tuberculosis, suggesting the potential impact of this polymorphism at the clinical level in lung tissue [8]. Based on these premises, we decided to evaluate the impact of P2X7 expression and polymorphisms in patients with non-small cell lung cancer (NSCLC).

## Results

### Patient characteristics

This study was conducted in 97 patients with NSCLC, including 50 with adenocarcinoma (ADC), 45 with squamous cell carcinoma (SCC), and 2 with large-cell carcinoma (LCC). The median age at diagnosis was 68 years (range: 46–85, mean: 67.37). Patients’ TNM classification was collected whenever available (80 cases) as follows: 12 T1, 45 T2, 17 T3, and 6 T4; and 26 cases with negative lymph node status, 49 with positive lymph node status (23 N1, 26 N2), and 5 with Nx status (Table 1).

**Table 1:** Correlations between P2X7 protein and the main clinico-pathological characteristics of the NSCLC patients

**Characteristic**	**P2X7 protein expression (%)**		***p***
	**Low**	**High**	
**Age**			
≤68 years	26 (50)	26 (50)	0.74
>68 years	24(53.3)	21(46.7)	
**Gender**			
Males	38(50.7)	37(49.3)	0.74
Females	12(54.5)	10(45.5)	
**Histology**			
ADC	27 (54)	23 (46)	0.88
SCC	22 (48.9)	23(51.1)	
LCC	1 (50)	1 (50)	
**Tumor stage**			
T1 (T1a-T1b)	4 (33.3)	8 (66.7)	0.08
T2 (T2a-T2b)	26(57.8)	19(42.2)	
T3	11(64.7)	6 (35.3)	
T4	1 (16.7)	5 (83.3)	
**Lymph-node status**			
Negative	11 (42.3)	15 (57.7)	0.08
Positive	31 (63.3)	18 (36.7)	
Nx	0 (0)	5 (100)	

### P2X7 immunohistochemistry

**Figure 1:**
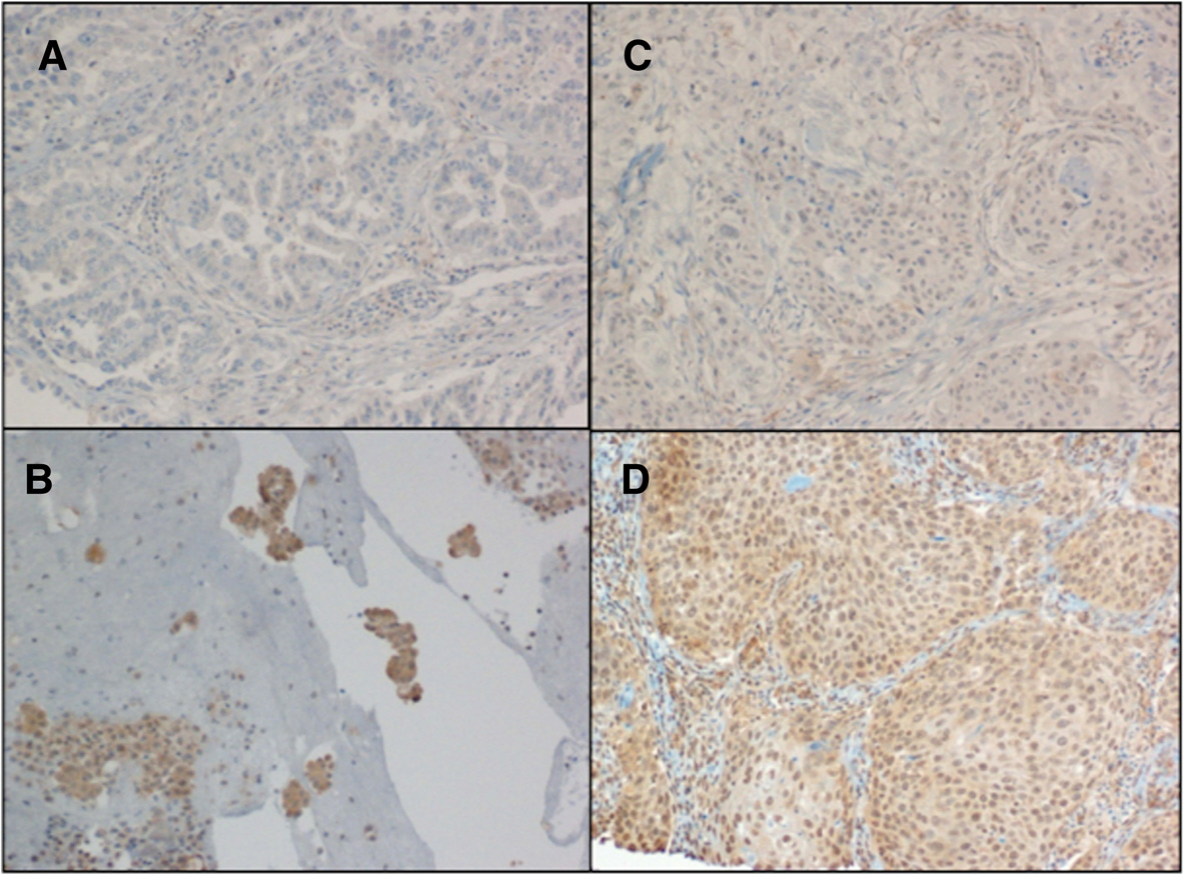
**Immunohistochemical staining of P2X7 in lung cancers.(A and C)** Low P2X7 expression levels in ADC (5%, 1+) and SCC (15%, 1+), respectively. **(B and D)** High P2X7 expression levels in ADC (80%, 2+) and SCC (70%, 2+), respectively. Original magnification, 10X.

Immunohistochemical expression of P2X7 was evaluated as the percentage of tumor cells displaying immunoreactivity. At least 1,000 cancer cells (100 cells in 10 HPFs) were counted for each section. The median value of P2X7 (30% of tumor positive cells) was used as a cut-off value to distinguish tumors with low P2X7 expression levels from those with high expression levels. There were 50 cases with low expression (11 negative, 17 with 10-15%, and 22 with 20-30% of tumor cells displaying immunoreactivity), while 47 samples showed high P2X7 immunohistochemical expression (30-90% immunoreactivity). The staining intensity was analyzed by distinguishing four categories: negative (0), weak staining (+), intermediate staining (++) and strong staining (+++). There was a good concordance between P2X7 percentage and intensity (chi-squared test, p = 0.01). Normal bronchial epithelial cells were used as internal positive controls for P2X7 staining. Negative controls were conducted by omitting the primary antibodies. Figure 1 shows representative P2X7 immunohistochemistry analyses in lung cancer.

### P2X7 polymorphism analysis

**Figure 2:**
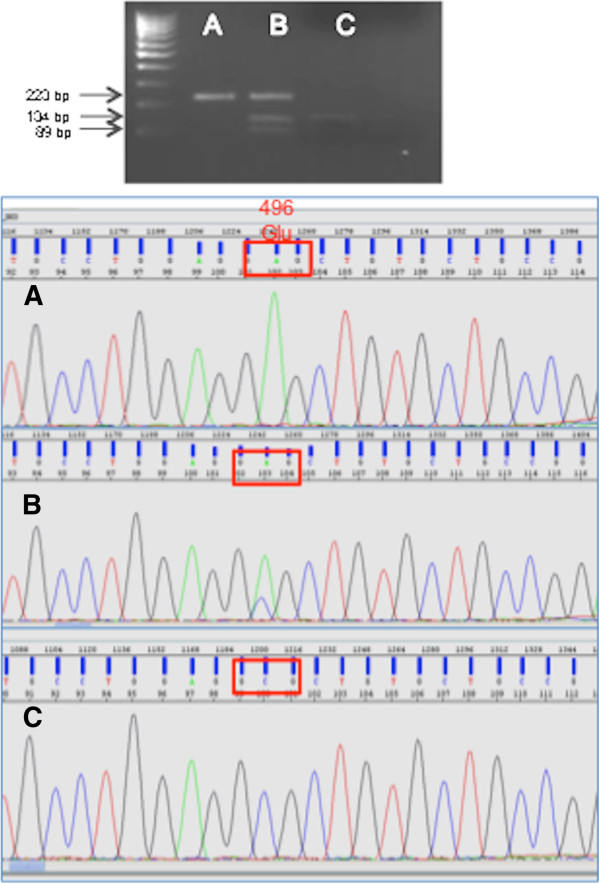
**P2X7 polymorphism. Upper panel**: The A1513C polymorphism of P2X7 was detected by restriction fragment length polymorphism (RFLP) analysis, as described in the Materials and Methods section. The PCR product was digested with *Hha*I and run on a 2% agarose gel. First lane, 100 bp marker; lane **A**, wild-type A/A (223 bp); lane **B**, heterozygote A/C (134 bp); lane C homozygote C/C (89 bp); last lane, negative control with no DNA added. **Lower panel**: sequencing analysis of PCR-RFLP products **A**, **B**, and **C** of the upper panel (wt, heterozygote and homozygote, respectively).

The A1513C SNP of P2X7 was genotyped by PCR/RFLP; Figure 2 shows a representative example. Regarding this polymorphism, the allelic frequencies of the A/A, A/C, and C/C genotypes in the whole cohort of NSCLC patients were 55.7% (54 of 97), 40.2% (39 of 97), and 4.1% (4 of 97), respectively. Therefore, the frequency of the minor allele was 0.2, resulting in Hardy-Weinberg equilibrium and a value within the range found in other published European reports [9].

We then analyzed minor allele frequency within the histotypes subgroups. No significant difference in the genotype frequency of the A1513C polymorphism was found between the two histological variants or among the main clinico-pathological characteristics of NSCLC patients (data not shown).

### P2X7 expression and clinico-pathological characteristics

We determined whether P2X7 expression was correlated with the main clinico-pathological characteristics. No statistically significant associations were observed between P2X7 protein expression and any of the main clinico-pathological characteristics of the NSCLC patients (Table 1).

## Discussion

P2X7 was originally thought to be restricted to cells of hematopoietic lineages. However, it is now evident that P2X7 is also present in other cell lineages and in various neoplastic cells, particularly those arising from hematopoietic and epithelial lineages [10,11]. P2X7 is overexpressed in several human cancers [12], conferring several key features of cancer cells. Moreover, P2X7 activation induces a number of well-established downstream signaling events, such as promoting cell proliferation or inducing cell death, via a number of different intracellular pathways. These observations raise the obvious issue of whether this receptor might be a useful marker as well as a novel target for therapy. Until now, few data existed regarding P2X7 expression in lung cancer. In this study, we have evaluated P2X7 expression in ninety-seven NSCLC samples by immunohistochemistry, evaluating the percentage of tumor cells displaying immunoreactivity. Malignant lung tissue showed P2X7 staining in most cases (86/97, 88.6%), with a good concordance between percentage and intensity of immunoreactivity. Then, we used the median value of tumoral positive cells as a cut-off to distinguish tumors with low P2X7 expression levels from those with high expression levels. No statistically significant associations were observed with the two histological variants of NSCLC, adenocarcinoma and squamous cell carcinoma, or with the main clinico-pathological characteristics of the lung cancer patients.

Polymorphic variants of P2X7 are also attracting considerable attention in human health and disease. P2X7 SNPs have been associated with various diseases. The robust association of SNP rs3751143 with increased susceptibility to tuberculosis [13] is most likely due to impaired P2X7-mediated killing of intracellular mycobacteria within macrophages [14,15]. However, the association of P2X7 SNPs with other diseases has been examined in a variety of other studies, with many failing to find significant associations [16]. The majority of these studies has small sample sizes and thus lacks sufficient statistical power to detect whether a significant association exists. In line with this finding, we also found in this study no significant difference in the genotype frequency of the A1513C polymorphism between the two histological variants or among the main clinico-pathological characteristics of NSCLC patients. Future studies examining the association of P2X7 SNPs with lung cancer will benefit from the development of high-throughput genotyping and the collection of large disease cohorts. Moreover, given the highly polymorphic nature of the P2X7 gene, future genetic association studies will need to include analysis of P2X7 haplotypes and the detailed phenotypic characterization of the encoded receptors.

It will also be of importance to determine the relative effectiveness of P2X7 therapeutics in relation to P2X7 isoforms and polymorphic variants in NSCLC. The role of P2X7 purinergic receptors in anticancer immune response and response to chemotherapy is not clear. Chemotherapy fails in the absence of the purinergic receptor in breast and colon cancer [17], but loss-of-function alleles in P2X7 do not affect overall survival in NSCLC patients, irrespective of the administration and type of chemotherapy [18]. A possible explanation for this difference is that bronchial carcinomas may be subjected to a less vigorous immunosurveillance than tumors located in other organs, such as the mammary gland or the colic mucosa.

## Conclusions

Based on our results, P2X7 expression and polymorphisms seem to have no potential impact in patients with non-small cell lung cancer; however, further studies, including micro-RNA-mediated gene regulation and other epigenetic mechanisms, will surely provide deeper insights into the results of our study.

## Materials and methods

### Patients

Ninety-seven NSCLC patients were retrospectively selected from patients operated at the Unit of Thoracic Surgery of the A.O.U.P. between 2005 and 2012. Histological diagnoses were independently formulated by two pathologists (G.F. and G.A.) according to the World Health Organization classification [19,20]. Clinico-pathological characteristics were collected in eighty cases. This study was conducted in compliance with the Helsinki Declaration and was approved by the Ethics Committee of the Institutuion. All of the patients gave their informed consent to analyses.

### P2X7 immunohistochemistry

Five-micrometer thick tumor sections were stained with anti-human P2X7 (Abcam, Cambridge, UK, 1:100 dilution) antibody in an automated immunohistochemistry processor (Immunostainer Benchmark XT; Ventana and Tuckson). For the detection of P2X7, sections were cut from formalin-fixed, paraffin-embedded blocks, deparaffinized with xylene, and rehydrated by sequential passages through decreasing (from 100% to 80%) concentrations of ethanol. Endogenous peroxidase activity was blocked by a 30-minute incubation at room temperature with methanol containing 3% H_2_O_2_. Tissue sections were then incubated at 98°C for 40 minutes in Target retrieval solution pH 9.0 (Dako); after several rinses in wash buffer (Dako), tissue sections were incubated overnight at 4°C with rabbit polyclonal anti-P2X7 antibody (ABCAM, 1:100). After incubation with the primary anti-antibody, tissue sections were rinsed twice in PBS and incubated for 30 minutes at room temperature with Dako Envision System HRP-conjugated rabbit antibodies. Tissue sections were then washed in PBS, and peroxidase activity was detected by incubation for 6 to 10 minutes at room temperature with Dako Liquid diaminobenzidine (DAB) Substrate Chromogen System (Dako). Counterstaining was conducted with Mayer's hematoxylin (Sigma-Aldrich).

### DNA isolation

After manual tumor macrodissection, DNA was isolated from 10-μm sections of formalin-fixed and paraffin-embedded (FFPE) tissue specimens using the QIAamp DNA Mini Kit (Qiagen), according to the manufacturer’s instructions.

### P2X7 polymorphism analysis

The A1513C SNP of P2X7 was genotyped by PCR/RFLP using the following primers: forward, 5′-TTCCTGGACAACCAGAGGAG-3′; reverse, 5′-AGGAACTGCAGGACGTGTCT-3′. Cycling conditions were as follows: 95°C for 4 min, 40 cycles of 95°C for 30 s, 58°C for 30 s and 72°C for 45 s, with a final 10-min extension at 72°C. PCR products were digested at 37°C for 16 h with 5.0 U of *HhaI* (Fermentas, Thermo Fisher Scientific, Milan, Italy). Digested products were run on a 2% agarose gel, which was stained with ethidium bromide and visualized using a UV transilluminator.

### Statistical analysis

One-way analysis of variance and chi-squared tests were used to determine the association between P2X7 expression and the polymorphism with the different parameters. Statistical analyses were performed using the JMP10 software, and a two-tailed p value <0.05 was considered significant.

## Abbreviations

NSCLC: Non-small cell lung cancer; ADC: Adenocarcinoma; SCC: Squamous cell carcinoma; LCC: Large-cell carcinoma.

## Competing interests

The authors declare that they have no competing interests.

## Authors’ contributions

LB designed the study, conducted data analysis and interpretation, and drafted the manuscript; MG carried out the molecular experiments; GA and GF formulated histological diagnoses; AS performed immunohistochemical assays; SP and CN participated in slide preparations; AM assisted in patient recruitment and clinic-pathological data collection. All authors read and approved the final manuscript.

## References

[B1] Adinolfi E Melchiorri L Falzoni S Chiozzi P Morelli A Tieghi A Cuneo A Castoldi G Di Virgilio F Baricordi OR P2X7 receptor expression in evolutive and indolent forms of chronic B lymphocytic leukemia Blood 2002 99 706 708 10.1182/blood.V99.2.706 11781259

[B2] Greig AV Linge C Healy V Lim P Clayton E Rustin MH McGrouther DA Burnstock G Expression of purinergic receptors in non-melanoma skin cancers and their functional roles in A431 cells J Invest Dermatol 2003 121 315 327 10.1046/j.1523-1747.2003.12379.x 12880424

[B3] Slater M Danieletto S Gidley-Baird A Teh LC Barden JA Early prostate cancer detected using expression of non-functional cytolytic P2X7 receptors Histopathology 2004 44 206 215 10.1111/j.0309-0167.2004.01798.x 14987223

[B4] Raffaghello L Chiozzi P Falzoni S Di Virgilio F Pistoia V The P2X7 receptor sustains the growth of human neuroblastoma cells through a substance P-dependent mechanism Cancer Res 2006 66 907 914 10.1158/0008-5472.CAN-05-3185 16424024

[B5] Solini A Cuccato S Ferrari D Santini E Gulinelli S Callegari MG Dardano A Faviana P Madec S Di Virgilio F Monzani F Increased P2X7 receptor expression and function in thyroid papillary cancer: a new potential marker of the disease? Endocrinology 2008 149 389 396 10.1210/en.2007-1223 17947359

[B6] Roger S Pelegrin P P2X7 receptor antagonism in the treatment of cancers Expert Opin Investig Drugs 2011 20 875 880 10.1517/13543784.2011.583918 21619470

[B7] Dardano A Falzoni S Caraccio N Polini A Tognini S Solini A Berti P Di Virgilio F Monzani F 1513A > C polymorphism in the P2X7 receptor gene in patients with papillary thyroid cancer: correlation with histological variants and clinical parameters J Clin Endocrinol Metab 2009 94 695 698 10.1210/jc.2008-1322 19017759

[B8] Fernando SL Saunders BM Sluyter R Skarratt KK Goldberg H Marks GB Wiley JS Britton WJ A polymorphism in the P2X7 gene increases susceptibility to extrapulmonary tuberculosis Am J Respir Crit Care Med 2007 175 360 366 10.1164/rccm.200607-970OC 17095747

[B9] Puxeddu E Moretti S Elisei R Romei C Pascucci R Martinelli M Marino C Avenia N Avenia N Rossi ED Fadda G Cavaliere A Ribacchi R Falorni A Pontecorvi A Pacini F Pinchera A Santeusanio F BRAF(V599E) mutation is the leading genetic event in adult sporadic papillary thyroid carcinomas J Clin Endocrinol Metab 2004 89 2414 2420 10.1210/jc.2003-031425 15126572

[B10] Deli T Csernoch L Extracellular ATP and cancer - an overview with special reference to P2 purinergic receptors Pathol Oncol Res 2008 14 219 231 10.1007/s12253-008-9071-7 18575829

[B11] White N Burnstock G P2 receptors and cancer Trends Pharmacol Sci 2006 27 211 217 10.1016/j.tips.2006.02.004 16530853

[B12] Di Virgilio F Ferrari D Adinolfi E P2X7: a growth-promoting receptor implications for cancer Purinergic Signal 2009 5 251 256 10.1007/s11302-009-9145-3 19263244 PMC2686832

[B13] Xiao J Sun L Yan H Jiao W Miao Q Feng W Wu X Gu Y Jiao A Guo Y Peng X Shen A Metaanalysis of P2X7 gene polymorphisms and tuberculosis susceptibility FEMS Immunol Med Microbiol 2010 60 165 170 10.1111/j.1574-695X.2010.00735.x 20846359

[B14] Saunders BM Fernando SL Sluyter R Britton WJ Wiley JS A loss-of-function polymorphism in the human P2X7 receptor abolishes ATP mediated killing of mycobacteria J Immunol 2003 171 5442 5446 10.4049/jimmunol.171.10.5442 14607949

[B15] Fernando SL Saunders BM Sluyter R Skarratt KK Wiley JS Britton WJ Gene dosage determines the negative effects of polymorphic alleles of the P2X7 receptor on adenosine triphosphate-mediated killing of mycobacteria by human macrophages J Infect Dis 2005 192 149 155 10.1086/430622 15942904

[B16] Sluyter R Stokes L Significance of P2X7 receptor variants to human health and disease Recent Pat DNA Gene Seq 2011 5 41 54 10.2174/187221511794839219 21303345

[B17] Zitvogel L Kepp O Kroemer G Immune parameters affecting the efficacy of chemotherapeutic regimens Nat Rev Clin Oncol 2011 8 151 160 10.1038/nrclinonc.2010.223 21364688

[B18] Vacchelli E Galluzzi L Rousseau V Rigoni A Tesniere A Delahaye N Schlemmer FD Menger L Sukkurwala AQ Adjemian S Martins I Michaud M Dunant A Kepp O Brambilla E Soria JC Zitvogel L Kroemer G Loss-of-function alleles of P2RX7 and TLR4 fail to affect the response to chemotherapy in non-small cell lung cancer Oncoimmunology 2012 1 271 278 10.4161/onci.18684 22737602 PMC3382853

[B19] Travis WD Brambilla E Muller-Hemerlink HK Harris CC World health organization classification of tumours. Pathology and genetics of tumours of the lung, pleura, thymus and heart 2004 Lyon, France: IARC Press

[B20] Travis WD Brambilla E Noguchi M Nicholson AG Geisinger KR Yatabe Y Beer DG Powell CA Riely GJ Van Schil PE Garg K Austin JH Asamura H Rusch VW Hirsch FR Scagliotti G Mitsudomi T Huber RM Ishikawa Y Jett J Sanchez-Cespedes M Sculier JP Takahashi T Tsuboi M Vansteenkiste J Wistuba I Yang PC Aberle D Brambilla C Flieder D Franklin W Gazdar A Gould M Hasleton P Henderson D Johnson B Johnson D Kerr K Kuriyama K Lee JS Miller VA Petersen I Roggli V Rosell R Saijo N Thunnissen E Tsao M Yankelewitz D International association for the study of lung cancer/American thoracic society/European respiratory society international multidisciplinary classification of lung adenocarcinoma J Thorac Oncol 2011 6 244 285 10.1097/JTO.0b013e318206a221 21252716 PMC4513953

